# Assessing the relationship between neighborhood factors and diabetes related health outcomes and self-care behaviors

**DOI:** 10.1186/s12913-015-1086-7

**Published:** 2015-10-01

**Authors:** Brittany L. Smalls, Chris M. Gregory, James S. Zoller, Leonard E. Egede

**Affiliations:** Center for Surgery and Public Health, Brigham and Women’s Hospital, 1620 Tremont Street, Suite 4-020, Boston, MA 02120 USA; Department of Healthcare Leadership and Management, College of Health Professions, Medical University of South Carolina, 151-B Rutledge Ave, MSC 962, Charleston, SC 29425 USA; Center for Health Disparities Research, Medical University of South Carolina, 135 Rutledge Ave, Room 280, MSC 593, Charleston, SC 29425 USA; Division of General Internal Medicine and Geriatrics, Department of Medicine, Medical University of South Carolina, 135 Rutledge Ave, RT 12th Floor, P.O. Box 250591, Charleston, SC 29425 USA

**Keywords:** Diabetes, Neighborhood/community, Social determinants, Self-care behaviors, Diabetes outcomes

## Abstract

**Background:**

Studies have shown that community and neighborhood characteristics can impact health outcomes of those with chronic illness, including T2DM. Factors, such as crime, violence, and lack of resources have been shown to be barriers to optimal health outcomes in diabetes. Thus, the objective of this study is to assess the effects of neighborhood factors on diabetes-related health outcomes and self-care behaviors.

**Methods:**

Adult patients (*N* = 615) with type 2 diabetes mellitus (T2DM) were recruited from an academic medical center and a Veterans Affairs medical center in the southeastern United States. Validated scales and indices were used to assess neighborhood factors and diabetes-related self-care behaviors. The most recent HbA1c, blood pressure, and LDL cholesterol were abstracted from each patients’ electronic medical record.

**Results:**

In the fully adjusted model, significant associations were between neighborhood aesthetics and diabetes knowledge (β = 0.141) and general diet (β = -0.093); neighborhood comparison and diabetes knowledge (β = 0.452); neighborhood activities and general diet (β = -0.072), exercise (β = -0.104), and foot care (β = -0.114); food insecurity and medication adherence (β = -0.147), general diet (β = -0.125), and blood sugar testing (β = -0.172); and social support and medication adherence (β = 0.009), foot care (β = 0.010), and general diet (β = 0.016). Significant associations were also found between neighborhood violence and LDL Cholesterol (β = 4.04), walking environment and exercise (β = -0.040), and social cohesion and HbA1c (β = -0.086).

**Discussion:**

We found that neighborhood violence, aesthetics, walking environment, activities, food insecurity, neighborhood comparison, social cohesion and social support have statistically significant associations with self-care behaviors and outcomes to varying degrees. However, the key neighborhood factors that had independent associations with multiple self-care behaviors and outcomes were food insecurity, neighborhood activities and social support.

**Conclusion:**

This study suggests that food insecurity, neighborhood activities, aesthetics, and social support may be important targets for interventions in individuals with T2DM.

**Electronic supplementary material:**

The online version of this article (doi:10.1186/s12913-015-1086-7) contains supplementary material, which is available to authorized users.

## Background

Type 2 diabetes mellitus (T2DM) affects more than 25.3 million people in the United States (US) and 366 million people worldwide [1–3]. Currently, there is an estimated 7 million undiagnosed cases of T2DM in the US [4]. By 2050, it is predicted that there will be 29 million individuals with T2DM in the US [5]. Major complications and comorbid illnesses attributed to T2DM, include blindness and vision problems, nervous system disorders, kidney disease, amputations, periodontal disease, heart disease, and stroke [1]. Consequently, T2DM is the seventh leading cause of death based on US death certificates in 2007 [1]. Also, in 2012, the estimated overall cost of T2DM in the US equaled $245 billion [6].

Social determinants of health are factors from the social environment that impact health of people and communities [5, 7]. Moreover, studies have shown that community and neighborhood characteristics can impact health outcomes of those with chronic illness, including T2DM [5, 8–10]. Factors, such as crime, violence, and lack of resources have been shown to be barriers to optimal health outcomes in diabetes [8, 9, 11–15]. Additionally, in a study conducted by Billimek and Sorkin [12], those who reported living in unsafe neighborhoods were 1.69-times more likely to report delays in filling prescriptions. Researchers have also evaluated neighborhood problems using the total score of patient’s rating of crime, trash, litter, lighting at night, access to exercise facilities, transportation and supermarkets [8]. Those with higher perceptions of neighborhood problems had higher rates of smoking, lower rates of physical activity, poor blood pressure control and lower scores on the SF-12 [8]. Even more compelling is a study done by Gary-Webb and colleagues [16] where combined known mediators, socioeconomic status and depression, and neighborhood characteristics were used to assess T2DM-related outcomes. This study evaluated the relationship between neighborhood-level socioeconomic status, health status and depression. The results of this study showed that individuals living in poverty had significantly lower scores for physical and emotional well being.

Though the results of the mentioned research are compelling, further research needs to be conducted to better understand these relationships. There is a need to better understand how different components of neighborhood and built environment impact multiple health outcomes in individuals with T2DM. The current study uses multiple validated scales and indices of neighborhood and community characteristics to identify which components of the neighborhood and community significantly impact health outcomes in individuals with T2DM. Therefore, the objective of this study is to assess the effects of neighborhood factors on multiple diabetes health outcomes, including HbA1c, blood pressure, and LDL cholesterol, and self-care behaviors. Based on current evidence, we hypothesized that 1) neighborhood problems, 2) neighborhood characteristics, 3) access to healthy foods and 4) social support will be significantly associated with diabetes-related health outcomes and self-care behaviors, after adjusting for relevant covariates.

## Methods

### Sample selection and setting

Patients with T2DM were recruited from an academic medical center and a Veterans affairs medical center in an urban location in southeastern United States. Approvals were obtained from the Medical University of South Carolina institutional review board and the Ralph H. Johnson Veterans Affairs Medical Center Research and Development Program prior to study enrollment. Eligible patients had to be 18 years of age or older, a patient at either facility with a diagnosis of T2DM in their medical record, and able to communicate in English. Subjects were ineligible if they exhibited mental confusion during the screening interview or reported alcohol or drug abuse/dependency using a validated CAGE-AID screening instrument [17].

### Data collection

Program Coordinators reviewed the electronic clinic roster to identify eligible patients. Eligible patients were approached in the clinic waiting room and provided a description of the study. Those interested and eligible were then consented and given a questionnaire, that inquired about various neighborhood factors as well sociodemographic information, to complete. Patients were able to complete the assessment before or after their scheduled clinic appointments, depending on clinic flow. Six hundred and fifteen participants were consented and completed the study. Study personnel who had direct contact with patients were required to conduct mock study visits with fellow study personnel to insure that the consent process and administration of the study assessment were standardized. Outcome measures (e.g., HbA1c, LDL cholesterol, and blood pressure) were abstracted from each patient’s electronic medical record.

### Outcome measures

#### Primary outcome measures

The primary outcome measure for this study was hemoglobin A1c (HbA1c) obtained within 6 months of the study visit and was abstracted from the electronic medical records.

#### Secondary outcome measures

The secondary outcome measures included blood pressure and low-density lipoprotein (LDL) cholesterol level. Blood pressure from the most recent visit and LDL within the past 12 months were abstracted from electronic medical records. The primary and secondary outcomes were selected based on guidelines for diabetes management by the American Diabetes Association.

### Neighborhood factors

#### Demographics

Patient demographics of interest included age, gender, race/ethnicity, marital status, household income, and health insurance. Age was categorized as 18–34 years, 35–44 years, 45–64 years, and 65 years and older. Race/ethnicity was categorized as non-Hispanic white, non-Hispanic black, and Hispanic/Other. Marital status was categorized as married or not married. Education was categorized as less than high school graduate, high school graduate, college, or graduate school. Employment was categorized as employed or not employed. Annual personal income was categorized as < $20,000, $20,000–$49,999, $50,000–74,999, and $75,000 and greater. Health insurance was categorized as insured or uninsured.

#### Social support

The Medical Outcomes Study (MOS) Social Support Survey [18] was used to measure social support. It measures four functional components of social support: 1) tangible support; 2) affection; 3) positive social interaction; and 4) emotional or informational support. The total scale (α = 0.97) and subscales (α = 0.91 to 0.96) have high internal consistency, good criterion and discriminant validity, and one-year test-retest reliability (0.72 to 0.76).

#### Neighborhood characteristics

Based on evidence from a prior validation study, six scales and four indices were used to assess neighborhood characteristics [19] (see Additional file 1). The six scales assessed aesthetic quality and consisted of 5 items, including questions about the neighborhood’s attractiveness, noise, and enjoyable scenery; walking/exercise environment and consisted of 11 items, such as heavy traffic, presence of trees for shade, easy to walk places; access to healthy foods and consisted of 11 items; crime/safety (safety had 3 items and crime had 4 items); and social cohesion and consisted of 5 items. The scales included items with response categories ranging from 1 (strongly agree) to 5 (strongly disagree). The violence scale response options ranged from 1 (often) to 4 (never). The recreation facilities index included 8 items on the presence of recreational facilities in the neighborhoods (yes/no) and rating of the facilities from 1 (excellent) to 4 (poor condition). Neighborhood participation index included 12 items that measure a person’s participation in civic and political activities with their neighbors. This index counts a “yes” response as 1 point and then summed the responses to indicate a person’s participation. Neighborhood problems index included 16 items measuring neighborhood characteristics such as presence of trash and litter. The response categories range from 1 to 3, with 1 indicating that the neighborhood attribute was not a problem, 2 it was somewhat of a problem, and 3 it was a big problem. Responses were summed to construct the neighborhood problems index score. For each of the scales and indices a higher score indicates more perceived problems in the neighborhood.

#### Food insecurity

This six-item scale constitutes the full set of adult items within the intermediate range of severity captured by the full food insecurity scale [20]. This set of questions has been shown to be the strongest available 6-item set across household with and without children, in relation to the full-scale-based classification of household food security status [20]. Items 1 and 2 are scored as affirmative if response is 1 often true or 2 sometimes true. They are scored as negative if response is 3 never true. Items 3, 5, and 6 are scored as affirmative if response is 1 (yes) and negative if response is 2 (no). Item 4 is scored affirmative if response is 1 (almost every month) or 2 (some months but not every month).

### Self-care behaviors

#### Self-reported medication adherence

This was measured with the 8-item self-report Morisky Medication Adherence Scale (MMAS) [21]. Each of the 8 items measures a specific medication-taking behavior. The new scale has higher reliability compared with the older 4-item scale (α = 0.83 vs. α = 0.61). The MMAS scores can range from 0 to 8 and was categorized as high adherence (score, 8), medium adherence (score, 6 to <8), and low adherence (score, <6).

#### Self-care behaviors

This was assessed with the Summary of Diabetes Self-Care Activities (SDSCA) scale [22]. SCDCA is a brief, validated self-report questionnaire of diabetes self-management that includes items assessing diet, exercise, medication adherence, and self blood glucose testing. The average inter-item correlations within scales are high; test-retest correlations are moderate; and correlations with other measures of diet and exercise generally support the validity of the SDSCA subscales.

### Statistical analyses

#### Sample size and power

The target sample size for the study was 600 adults with T2DM. With 600 participants, the analysis to evaluate the univariate relationships between community/neighborhood characteristics and diabetes care processes and outcomes would have 80 % power to detect an association of at least ρ = 0.3, where ρ represents the population correlation between the dependent (i.e. diabetes processes and outcomes) and each primary independent variable. For the multivariate analyses involving evaluation of the relationship between diabetes processes and outcomes and each primary independent variable adjusted for covariables (e.g., age, sex, education, income, marital status, and employment), the study would be able to detect with 80 % power an increment of at least 10 % in R^2^ for a given primary independent variable, over and above the contribution of the covariables. Specifically, the increment in R^2^ represents the proportion of variation in the outcome variable accounted for by each primary independent variable over and above that explained by the covariables. As defined by Cohen [23] we will have 80 % power to detect between a small effect (primary independent variable accounts for 2 % of the variance of the dependent variable) and a moderate effect (primary independent variable accounts for 13 % of the Y variance).

#### Data analyses

The conceptual model for this paper is based on the work of Brown and colleagues [9]. This model begins with an individual’s socioeconomic position, which includes education, income, employment, and community crime rates among other characteristics. The model posits that any component of an individual’s socioeconomic position over the course of their life could contribute to health outcomes as an adult. This model hypothesizes that socioeconomic position has an impact on T2DM health outcomes. Socioeconomic position is characterized by an individual’s education level, employment status, and occupational prestige; the level of wealth or income experienced as a child and as an adult; and community/neighborhood factors, such as income, education, and crime rates. Subsequently, socioeconomic position influences proximal moderators/mediators, including health behaviors, access to care, and process of care. Additionally, there are distal moderators/mediators that take into account an individual’s stress level, provider decision-making style, community availability of health foods, and the health care system. As a result, socioeconomic position and distal and proximal moderators/mediators are thought to have an impact on health outcomes (e.g., health status, quality of life, glycemic control). For this analysis, we propose that neighborhood characteristics are composed of: (1) access to healthy foods and food insecurity, (2) walking and exercise environment, (3) social cohesion and social support, and (4) aesthetic environment and neighborhood quality.

We performed three sets of analyses. First, we compared sample demographics using chi square statistics for categorical variables and t-test or one-way analysis of variance (ANOVA) for continuous variables. Second, we ran multiple regression models to assess the independent associations between neighborhood factors and diabetes knowledge and self-care behaviors (medication adherence, diet, physical activity, blood sugar testing and foot care) controlling for covariates. For each regression model, diabetes knowledge or self-care behaviors (medication adherence, diet, physical activity, blood sugar testing and foot care) were the dependent variables, neighborhood factors were the primary independent variables and age, sex, race, education, employment, income, marital status, health literacy, quality of life, and comorbidities were included in the model as covariates. Variables were entered in blocks based on theoretical relationships. The sequence of entry were as follows: 1) neighborhood problems, 2) neighborhood characteristics, 3) access to healthy foods, 4) social support, and 5) sociodemographic variables. The final model included all neighborhood factors adjusting for covariates. Third, we ran multiple regression models to assess the independent effect of neighborhood factors on glycemic control, systolic blood pressure and LDL cholesterol controlling for covariates. For the regression models, we used age category 45-64 years and the college education category as our reference groups because they included the majority of the sample population. In this model, HbA1c, systolic blood pressure, and LDL cholesterol were the dependent variables, neighborhood factors were the primary independent variables and the same covariates as above were included. Similar to the approach above, variables were entered in blocks based on theoretical relationships. The final model included all neighborhood factors adjusting for covariates. Finally, for each of the multiple regression models, we assessed standardized betas for each variable in the model to determine the amount of variance explained by the neighborhood factors. All analyses were performed with STATA V13 and a two tailed alpha of 0.05 was used to assess for significance.

## Results

Demographics and neighborhood characteristics, for this sample of 615 adults with type 2 diabetes are shown in Tables 1 and 2. The majority of these patients were men (61.6 %), non-Hispanic Black (65.7 %), between the ages of 45-64 (53.6 %), not employed (65.3 %), were college educated (47.1 %), had an income less than $20,000 annually (41.6 %), and were insured by Medicare (24.8 %). Also, the majority of this population had blood pressure less than 140/80 mmHg (58.9 %), LDL cholesterol less than 100 mg/dL (63.9 %), had an HbA1c greater than 7 % (60.9 %), and had a body mass index greater than 30. In general, participants reported low to moderate crime, violence, walkability, and aesthetic environments. However, participants reported higher scores for social cohesion, social support, participating in neighborhood activities, neighborhood rating and comparison between participants’ neighborhood and other neighborhoods within the same county.

**Table 1:** Sample demographic characteristics (*n* = 615)

Age	
18–34 years	1.6
35–44 years	5.2
45–64 years	53.6
65+ years	39.6
Gender	
Women	38.4
Men	61.6
Race/ethnicity	
Non-Hispanic Black	65.7
Non-Hispanic Whites	33.0
Hispanic/Other	1.3
Marital status	
Married	49.7
Not married	50.3
Educational level	
Less than high school graduate	13.0
High school graduate	28.2
College education	47.1
More than college	11.7
Employment status	
Employed	34.7
Not employed	65.3
Annual income level	
<$20,000	41.6
$20,000–$49,000	38.9
$50,000–$74,999	10.1
$75,000+	9.4
Health insurance	
Uninsured	9.12
Private	20.2
Medicare	24.8
Medicaid	10.3
Military/TRICARE	23.9
Other	11.7
Blood pressure control (<140/80 mmHg)	
Controlled	58.9
Not controlled	41.1
Lipid control (LDL < 100 mg/dL)	
Controlled	63.9
Not controlled	36.1
Glycemic control (HbA1c < 7 %)	
Controlled	39.1
Not controlled	60.9
Body Mass Index (>30)	68.2

**Table 2:** Summary of participants’ neighborhood characteristics (*n* = 615)

Neighborhood characteristics	Mean (±SD)
Aesthetic environment (5–25)^a^	15.2 (±2.75)
Walking environment (11–55)^a^	28.6 (±7.11)
Safety from crime (3–15)	8.49 (±2.04)
Access to healthy foods (6–30)	16.2 (±7.21)
Social cohesion (5–25)^a^	14.2 (±2.47)
Social support (0–100)^a^	72.8 (±26.1)
Neighborhood violence (4–16)	5.15 (±1.97)
Presence of crime (1–4)	2.06 (±0.81)
Neighborhood rating (1–4)	1.8 (±0.76)
Neighborhood comparison (1–5)	2.1 (±0.91)
Recreational facilities index (0–8)^a^	4.4 (±2.49)
Neighborhood participation index (0–12)^a^	8.9 (±3.24)
Neighborhood problems index (0–16)	12.4 (±3.71)
Food insecurity (0–6)	1.3 (±1.93)

^a^Higher score is better

Table 3 shows the final model of the relationship between neighborhood factors and self-care behaviors and diabetes knowledge. The final model showed a statistically significant (*p* < 0.05) relationship between participating in neighborhood activities and adhering to a general diet (β = -0.072, CI 95 %: -0.133, -0.011), exercise (β = -0.104, CI 95 %: -0.171, -0.038), and foot care (β = -0.115, CI 95 %: -0.190, -0.039); neighborhood aesthetics and diabetes knowledge (β = 0.141, CI 95 %: 0.012, 0.269) and general diet (β = -0.093, CI 95 %: -0.166, -0.020); neighborhood comparison and diabetes knowledge (β = 0.452, CI 95 %: 0.036, 0.867); food insecurity and medication adherence (β = -0.147, CI 95 %: -0.243, -0.052), general diet (β = -0.124, CI 95 % -0.224, -0.025), and blood sugar testing (β = -0.172, CI 95 %: -0.298, -0.046); and social support and medication adherence (β = 0.009, CI 95 % 0.002, 0.016), general diet (β = 0.016, CI 95 %: 0.009, 0.023), and foot care (β = 0.010, CI 95 %: 0.001, 0.019).

**Table 3:** Final models of relationship between neighborhood factors on knowledge and self care behaviors

	Diabetes knowledge	Medication adherence	General diet	Exercise	Blood sugar testing	Foot care
Neighborhood problems						
Safety	−0.016	0.019	0.0002	−0.097	0.002	0.007
Violence	0.001	−0.089	−0.036	0.007	−0.092	0.015
Crime	−0.341	0.220	0.119	0.114	0.209	0.156
Problems	−0.017	−0.012	0.004	−0.026	−0.001	0.074
Neighborhood characteristics						
Aesthetics	0.141*	0.042	−0.093*	−0.005	−0.053	−0.084
Walking environments	0.010	−0.019	0.014	−0.040*	0.034	0.036
Recreational facilities	0.061	0.040	0.025	−0.054	−0.001	−0.020
Neighborhood activities	0.007	−0.018	−0.072*	−0.104*	−0.057	−0.114*
Neighborhood rating	−0.137	0.017	−0.052	0.058	−0.107	0.064
Neighborhood comparison	0.452*	−0.224	−0.082	−0.153	−0.086	0.024
Access to healthy food						
Food insecurity	−0.108	−0.147*	−0.125*	−0.106	−0.172*	−0.046
Access to healthy food	−0.036	−0.025	−0.019	0.018	−0.007	−0.016
Social support						
Social cohesion	0.090	0.046*	0.028	0.026	−0.019	0.070
Social support	0.001	0.009*	0.016**	0.006	0.009	0.010*
Comorbidities	0.040	−0.132*	0.048	−0.064	0.191**	0.173**
Health literacy						
Low (ref)						
Marginal	0.025	0.714	0.539	0.338	0.158	0.524
Adequate	2.12**	0.494	0.412	0.258	0.350	0.475
Age						
18–34 years	−0.384	−0.801	−0.987	−0.501	−0.191	1.58
35–44 years	−0.187	−0.230	0.120	0.281	0.287	−0.437
45–64 (ref)						
65+ years	−0.614	0.507*	0.529*	0.182	0.156	0.215
Gender						
Male (ref)						
Female	0.277	0.103	−0.216	−0.265	−0.269	0.015
Marital status						
Never married (ref)						
Married	0.312	0.514	−0.156	−0.356	−0.328	0.463
Separated/divorced	−0.195	0.419	0.218	−0.174	−0.576	0.221
Widowed	−0.152	0.208	−0.055	−0.546	−0.526	0.816
Race						
Non-Hispanic White (ref)						
Non-Hispanic Black	−0.509	−0.335	0.065	−0.087	0.223	0.516*
Hispanic/other	0.598	−2.16*	−1.50	−0.812	−0.593	−1.28
Education						
<High school	−1.76*	0.017	0.128	−0.431	−0.707	0.054
High school	−0.782*	0.009	−0.199	−0.055	−0.277	−0.031
College (ref)				0.544		
Graduate school	0.650	−0.17	0.028		−0.561	0.193
Employment						
Unemployment (ref)						
Employed	−0.600	−0.447*	−0.052	0.320	−0.138	0.053
Income						
<$19,999 (ref)						
$20,000–49,999	0.482	−0.079	0.029	−0.779*	−0.453	−0.164
$50,000–74,000	1.15	−0.506	−0.304	−0.712	−1.02	0.584
≥$75,000	0.802	−0.206	−0.137	−0.565	−0.771	−0.848
Health insurance						
Uninsured (ref)						
Private	0.361	0.401	−0.178	−0.723	0.533	−0.398
Medicare	−0.159	0.138	−0.657	−0.702	0.225	−0.498
Medicaid	-.0737	0.999*	−0.261	−0.126	1.35*	0.873
Military	−0.082	−0.026	−0.820*	−0.121	0.235	0.139
Other insurance	−0.643	0.590	0.043	−0.617	0.576	0.358
R^2^	0.208	0.155	0.096	0.101	0.043	0.082

*p < 0.05, **p < 0.001, ref = reference group

Final model for each outcome after hierarchical models run entering variables in blocks based on theoretical relationships – first model included variables characterized as neighborhood problems, second block added variables characterized as neighborhood characteristics, third block added variables, characterized as access to healthy foods, fourth block added variables characterized as social support, and fifth block added sociodemographic variables

In addition, we ran standardized betas to estimate the amount of variance in self-care behaviors that was explained by neighborhood factors. For medication adherence, 14.2 % was explained by food insecurity and 11.6 % was explained by social support; for general diet, 20.3 % was explained by social support, 12.6 % was explained by neighborhood aesthetics, and 11.5 % by neighborhood activities and food insecurity, respectively; for blood sugar testing, 13.3 % was explained by food insecurity; for exercise, 12.7 % was explained by walking environment and 15.2 % by neighborhood activities; diabetes knowledge was explained by 10.1 % of neighborhood aesthetics and 10.7 % by neighborhood comparison; and for foot care, 14.9 % was explained by neighborhood activities and 10.7 % was explained by social support.

Table 4 shows the final model of the relationship between neighborhood factors and diabetes-related health outcomes. The final model showed a statistically significant (*p* < 0.05) relationship between neighborhood violence and LDL cholesterol (β = 4.04, CI 95 %: 0.149, 7.93), and social cohesion and glycemic control (β = -0.086, CI 95 %: -0.156, -0.015).

**Table 4:** Final models of relationship between neighborhood factors on diabetes outcomes and quality of life

	HbA1c	Systolic blood pressure	LDL cholesterol
Neighborhood problems			
Safety	0.019	−0.058	−0.116
Violence	0.053	0.429	4.04*
Crime	0.105	−0.048	3.54
Problems	0.045	−0.331	0.658
Neighborhood characteristics			
Aesthetics	0.054	−0.608	1.67
Walking environments	0.011	0.160	−0.654
Recreational facilities	0.016	−0.101	−0.368
Neighborhood activities	−0.017	0.166	1.37
Neighborhood rating	0.163	−1.28	−0.412
Neighborhood comparison	−0.006	0.504	−3.89
Access to healthy food			
Food insecurity	0.092	0.226	−1.87
Access to healthy food	−0.001	−0.126	−0.355
Social support			
Social cohesion	−0.086*	0.295	−0.385
Social support	−0.002	0.011	−0.132
Comorbidities	0.005	−0.045	0.737
Health literacy			
Low (ref)			
Marginal	−0.326	2.88	−13.9
Adequate	−0.230	3.52	−7.31
Age			
18–34 years	−0.029	2.07	1.71
35–44 years	0.395	−1.62	−17.5
45–64 years (ref)			
65+ years	−0.363	2.45	−10.7
Gender			
Male (ref)			
Female	−0.239	3.58*	15.8*
Marital status			
Never married (ref)			
Married	0.179	0.807	16.8
Separated/divorced	0.163	1.86	13.4
Widowed	−0.240	0.820	22.6
Race			
Non-Hispanic White (ref)			
Non-Hispanic Black	−0.112	4.10	9.21
Hispanic/other	0.170	3.35	30.2
Education			
<High school	0.238	5.02	3.23
High school	−0.134	0.972	3.95
College (ref)			
Graduate school	−0.221	4.57	14.6
Employment			
Unemployment (ref)			
Employed	0.087	−1.12	0.448
Income			
<$19,999 (ref)			
$20,000–49,999	0.098	3.45	3.89
$50,000–74,000	0.188	2.58	11.9
≥$75,000	−0.382	8.15*	−6.54
Health insurance			
Uninsured (ref)			
Private	−0.106	2.53	−3.26
Medicare	−0.234	1.59	−23.3
Medicaid	−0.686	6.51	−23.6
Military	−0.185	−1.64	−14.6
Other insurance	−0.182	−4.28	−28.4*
R^2^	0.041	0.042	0.016

* *p* < 0.05, ref = reference group

Final model for each outcome after hierarchical models run entering variables in blocks based on theoretical relationships – first model included variables characterized as neighborhood problems, second block added variables characterized as neighborhood characteristics, third block added variables, characterized as access to healthy foods, fourth block added variables characterized as social support, and fifth block added sociodemographic variable

Similarly, we ran standardized betas to estimate the amount of variance in outcomes that was explained by neighborhood factors. For LDL cholesterol, 12.6 % was explained by violence and for glycemic control, 10.6 % was explained by social cohesion.

Table 5 summarizes the significant associations. Food insecurity was significantly associated with HbA1c, medication adherence, diet and blood sugar testing; neighborhood activities were significantly associated with diet, exercise and foot care; while social support was significantly associated with medication adherence, diet and foot care; violence was significantly associated with LDL cholesterol; neighborhood aesthetics were significantly associated with diabetes knowledge and diet; walking environment was significantly associated with exercise; neighborhood comparison was significantly associated with diabetes knowledge; and social cohesion was significantly associated with glycemic control. This suggests that several neighborhood factors are important targets for interventions in individuals with T2DM.

**Table 5:** Significant neighborhood factors for each modeled outcome

Outcome variable	HbA1c	LDL	Systolic blood pressure	Diabetes knowledge	Medication adherence	General diet	Exercise	Blood sugar testing	Foot care
Safety									
Violence		X							
Crime									
Problems									
Aesthetics				X		X			
Walking environment							X		
Recreational facilities									
Neighborhood participation						X	X		X
Neighborhood rating									
Neighborhood comparison				X					
Food insecurity					X	X		X	
Access to healthy food									
Social cohesion	X								
Social support					X	X			X

Significance based on *p* < 0.05 within final model for each outcome. Neighborhood factors were noted as significant if any category showed significance in final model

## Discussion

This study adds to the current literature by using multiple validated scales and indices that measured neighborhood and community characteristics and examined their independent relationships with self-care and outcomes in patients with T2DM. We found that neighborhood violence, aesthetics, walking environment, activities, food insecurity, neighborhood comparison, and social support have statistically significant associations with diabetes self-care behaviors and health outcomes to varying degrees. However, we did not find significant relationships between neighborhood safety, crime, perceived neighborhood problems, availability of recreational facilities, neighborhood rating, and access to healthy foods and self-care behaviors and health outcomes. This is the first study to our knowledge that used a large sample size and all available measures of neighborhood factors to assess these relationships. In addition, we entered variables in blocks and examined the amount of variance explained by each significant neighborhood and community factor. This study provides new evidence on significant neighborhood factors and potential targets for future intervention.

Our findings are consistent with the work of Brown et al. [9], Kollannoor-Samuel et al. [11], Solais et al. [14] and Gariepy et al. [10] but contradict the findings from some prior studies of Gary et al. [8] and Bellimek et al. [12]. We found that the key neighborhood factors that had independent associations with multiple self-care behaviors and outcomes were food insecurity (significantly associated with HbA1c, medication adherence, diet and blood sugar testing), neighborhood activities (significantly associated with diet, exercise and foot care) and social support (significantly associated with diet and foot care). This suggests that food insecurity, neighborhood activities and social support may be important targets for interventions in individuals with T2DM.

It is important to note that glycemic control, the primary outcome variable for T2DM, was only significantly associated with social cohesion. This further strengthens the evidence that suggests social cohesion and social support influences health and health outcomes [24–27]. Research has also shown that there is an association between social cohesion and social support and other health risk factors, such as chronic illness, mortality and poor self-management behaviors [28]. Similarly, participating in and the availability of neighborhood activities were associated with self-care behaviors. It can be argued that social cohesion, social support, and neighborhood activities share common attributes that may predict self-care behaviors in patients with T2DM. Additionally, prior studies have documented that food insecurity has negative impact on health [11, 29–33].

The literature has suggested that neighborhood characteristics have direct and indirect effects, via self-care behaviors, on diabetes related health outcomes [34]. Still, additional work is needed to further understand the nuances that underlie the relationship between social cohesion/social support, neighborhood activities, and food insecurity and diabetes self-care behaviors and outcomes.

However, when considering clinical significance of the relationship between neighborhood characteristic and clinically relevant health outcomes the relationships are telling. Glycemic control has a positive association with all the neighborhood characteristics except neighborhood activities, neighborhood comparison, access to healthy foods, social cohesion, and social support. Systolic blood pressure was also positively associated with most the neighborhood characteristics, excluding safety, perceived neighborhood problems, aesthetics, recreational facilities, neighborhood rating, and access to healthy foods. The other outcome of interest, LDL cholesterol, also had positive associations with the exception of walking environment, recreational facilities, neighborhood rating, neighborhood comparison, food insecurity, access to healthy foods, social cohesion, and social support. Though not all of these relationships were statistically significant, they have clinical relevance because these health outcomes not only impact diabetes health outcomes, potentially increasing the likelihood of developing comorbid conditions. Moreover, similar associations can be seen between neighborhood characteristics and self-care behaviors, which are believed to have a mediating/moderating role in diabetes health outcomes.

The strengths of the current study include use of validated theoretical and conceptual models, large sample size, inclusion of a broad range of neighborhood and community factors, entry of variables in blocks based on theoretical relationships to assess incremental effect, and focus on multiple self-care behaviors and outcomes. However, the study has some limitations. First, as with all cross-sectional studies, we cannot speak to direction or causality. We cannot determine with certainty whether neighborhood characteristics are the cause of poor health outcomes or if those with poor health outcomes have poor neighborhood characteristics, although our theoretical model suggest that neighborhood factors are in the pathway to self-care and outcomes. Second, the study population was a convenience sample from the southeastern United States and may not be representative of individuals with diabetes from other parts of the country. Third, we did not ask patients the duration of time they had lived in the community for which they were reporting. Finally, there may be other important covariates that were not included in our study; however, we based our variable selection on an established theoretical/conceptual model, which strengthens our findings.

## Conclusion

In conclusion, we found that neighborhood violence, aesthetics, activities, food insecurity, neighborhood comparison, walking environment and social cohesion/social support have statistically significant associations with diabetes self-care behaviors and health outcomes to varying degrees and that key neighborhood factors that had independent associations with multiple self-care behaviors and outcomes were food insecurity, neighborhood activities and social support. This suggests that food insecurity, neighborhood activities and social support may be important targets for future interventions in individuals with T2DM.

## Additional file

Additional file 1:
**Neighborhood Characteristics Assessment.** (DOC 1603 kb)
