# Supplementary material for: Assessing the relationship between neighborhood factors and diabetes related health outcomes and self-care behaviors
Source: BMC Health Serv Res. 2015 Oct 1;15:445. doi: 10.1186/s12913-015-1086-7 (PMC4589943; doi:10.1186/s12913-015-1086-7)
Supplement: Additional file 1: — Neighborhood Characteristics Assessment. (DOC 1603 kb) [file 12913_2015_1086_MOESM1_ESM.doc]

**Demographics** Page 1 of 1

| 1 | Date of Birth | - -   (DD-MMM-YYYY) |
| --- | --- | --- |
| 2 | Age |  |
| 3 | Sex | Female Male |
| 4 | Marital Status | Never Married  Married  Separated/Divorced  Widowed |
| 5 | Race | White Hispanic  Black Other: |
| 6 | How long have you had diabetes |  |
| 7 | How often do you have difficulty understanding or speaking English? | Always  Often  Sometimes  Rarely  Never  Don’t know |
| 8 | How often do you have difficulty reading or writing English? | Always  Often  Sometimes  Rarely  Never  Don’t know |

| General Comments: |
| --- |

**Social Economic and Health Status** Page 1 of 2

| 1 | How many years of formal education beyond kindergarten did you complete?  (example: 1 year of college = 13 years) | years of schooling |
| --- | --- | --- |
| 2 | How many hours do you work per week?  (if none, enter 0) | hours/week |
| 3 | What is your current employment status or activity? | Working for pay (full-time or part time, includes self-employment)  Unable to work due to poor health or disability  Unemployed, laid-off or on strike  Student  Fulltime homemaker, parent or caregiver  Retired: what year? ______  Other (specify) _______________  Don’t know |
| 4 | Which of these categories best describes your annual household income from all sources? | Less than $10,000    $10,000 - < $15,000    $15,000 - < $20,000    $20,000 - < $25,000    $25,000 - < $35,000    $35,000 - < $50,000    $50,000 - < $75,000    $75,000 or more |

**Social Economic and Health Status** Page 2 of 2

| 5 | How would you describe the insurance plan(s) you have had in past 12months | None-no insurance  Private Insurance  Medicare  Medicaid  Military Care  Other insurance |
| --- | --- | --- |
| 6 | In general, would you say your health is: (choose one) | 1 Excellent  2 Very Good  3 Good  4 Fair  5 Poor |
| 7 | Do you have any health problem that requires you to use special equipment (such as a cane, wheelchair, special bed, or special telephone)? | Yes No |
| 8 | Have you smoked at least 100 cigarettes in your entire life? | Yes No |
| 9 | How often do you NOW smoke cigarettes? | Every day  Some days  Not at all |
| 10 | How many days in a week do you do MODERATE TO VIGOROUS activities for AT LEAST 20 MINUTES that cause HEAVY sweating or LARGE increases in breathing or heart rate? | Never 1-2 days  3-4 days 5 or more days |

| General Comments: |
| --- |

**The Medical Outcomes Study (MOS) Social Support Survey Form Page 1 of 2**

| People sometimes look to others for companionship, assistance or other types of support.  How often is each of the following kinds of support available to you if you need it?  Choose one number on each line. |
| --- |

| **Emotional/informational support** | | None of the time | A little of the time | Some of the time | Most of the time | All of the time |
| --- | --- | --- | --- | --- | --- | --- |
| 1 | Someone you can count on to listen to when you need to talk | 1 | 2 | 3 | 4 | 5 |
| 2 | Someone to give you information to help you understand a situation | 1 | 2 | 3 | 4 | 5 |
| 3 | Someone to give you good advice about a crisis | 1 | 2 | 3 | 4 | 5 |
| 4 | Someone to confide in or talk to about yourself or your problems | 1 | 2 | 3 | 4 | 5 |
| 5 | Someone whose advice you really want | 1 | 2 | 3 | 4 | 5 |
| 6 | Someone to share your most private worries and fears with | 1 | 2 | 3 | 4 | 5 |
| 7 | Someone to turn to for suggestions about how to deal with a personal problem | 1 | 2 | 3 | 4 | 5 |
| 8 | Someone who understands your problems | 1 | 2 | 3 | 4 | 5 |
| **Tangible Support** | |  | | | | |
| 9 | Someone to help you if you were confined to bed | 1 | 2 | 3 | 4 | 5 |
| 10 | Someone to take you to the doctor if you needed it | 1 | 2 | 3 | 4 | 5 |
| 11 | Someone to prepare your meals if you were unable to do it yourself | 1 | 2 | 3 | 4 | 5 |
| 12 | Someone to help with daily chores if you were sick | 1 | 2 | 3 | 4 | 5 |

**The Medical Outcomes Study (MOS) Social Support Survey Form**  Page 2 of 2

| **Affectionate support** | | None of the time | A little of the time | Some of the time | Most of the time | All of the time |
| --- | --- | --- | --- | --- | --- | --- |
| 13 | Someone who shows you love and affection | 1 | 2 | 3 | 4 | 5 |
| 14 | Someone to love you and make you feel wanted | 1 | 2 | 3 | 4 | 5 |
| 15 | Someone who hugs you | 1 | 2 | 3 | 4 | 5 |
| **Positive Social Interaction** | |  | | | | |
| 16 | Someone to have a good time with | 1 | 2 | 3 | 4 | 5 |
| 17 | Someone to get together with for relaxation | 1 | 2 | 3 | 4 | 5 |
| 18 | Someone to do something enjoyable with | 1 | 2 | 3 | 4 | 5 |
| **Additional Item** | |  | | | | |
| 19 | Someone to do things with to help you get your mind off things | 1 | 2 | 3 | 4 | 5 |
| General Comments: | | | | | | |

**Diabetes Knowledge Questionnaire**  Page 1 of 2

| 1 | Eating too much sugar and other sweet food is a cause of diabetes | Yes No I don’t know |
| --- | --- | --- |
| 2 | The usual cause of diabetes is lack of insulin in the body | Yes No I don’t know |
| 3 | Diabetes is caused by failure of the kidneys to keep sugar out of the urine | Yes No I don’t know |
| 4 | Kidneys produce insulin | Yes No I don’t know |
| 5 | In untreated diabetes, the amount of sugar in the blood usually increases | Yes No I don’t know |
| 6 | If I am a diabetic, my children have a higher chance of being a diabetic | Yes No I don’t know |
| 7 | Diabetes can be cured. | Yes No I don’t know |
| 8 | A fasting blood sugar of 210 is too high. | Yes No I don’t know |
| 9 | The best way to check my diabetes is by testing my urine. | Yes No I don’t know |
| 10 | Regular exercise will increase the need for insulin or other diabetic medication | Yes No I don’t know |
| 11 | There are two main types of diabetes: Type 1 (insulin dependent) and Type 2 (non-insulin dependent) | Yes No I don’t know |
| 12 | An insulin reaction is caused by too much food. | Yes No I don’t know |
| 13 | Medication is more important than diet and exercise to control my diabetes | Yes No I don’t know |

**Diabetes Knowledge Questionnaire Page 2 of 2**

| 14 | Diabetes often causes poor circulation. | Yes No I don’t know |
| --- | --- | --- |
| 15 | Cuts and abrasions on diabetics heal more slowly. | Yes No I don’t know |
| 16 | Diabetics should take extra care when cutting their toenails. | Yes No I don’t know |
| 17 | A person with diabetes should cleanse a cut with iodine and alcohol. | Yes No I don’t know |
| 18 | The way I prepare my food is as important as the food I eat. | Yes No I don’t know |
| 19 | Diabetes can damage my kidneys. | Yes No I don’t know |
| 20 | Diabetes can cause loss of feeling in my hands, fingers, and feet. | Yes No I don’t know |
| 21 | Shaking and sweating are signs of high blood sugar. | Yes No I don’t know |
| 22 | Frequent urination and thirst are signs of low blood sugar. | Yes No I don’t know |
| 23 | Tight elastic hose or socks are not bad for diabetics. | Yes No I don’t know |
| 24 | A diabetic diet consists mostly of special foods. | Yes No I don’t know |

**TOFHLA** Page 1 of 1

| **INSTRUCTIONS FOR TOFHLA**  Now, I’d like to check your ability to read and understand the things doctors often give patients, like medical instructions. Many people have a hard time understanding medical instructions, and this can affect your health.  Do you wear glasses? (IF YES….) Please put them on.  I am going to show you some medical instructions that you might see around a hospital. There are words missing in these instructions, like in this example (HAND EXAMPLE). I want you to fill in the missing words. Just below the blank line, we’ve listed 4 possible words to complete the sentence. Choose the word that makes the most sense. Circle the letter in front of the word you choose. So, in this example, “Your blood test was blank”, the word “normal” is the only word that can complete the sentence so it makes sense. So, you would circle the letter “a” in front of the word “normal”. Is that clear?  **EXAMPLE**  Your blood test was __________  a. normal  b. take  c. hurt  d. germs  There are four pages, and the page numbers are shown at the bottom of each page. **TURN TO FIRST PAGE AND SHOW NUMBER.** Be careful not to skip any. You will have 7 minutes to complete this. Just complete as much as you can in that time. |
| --- |

**TOFHLA**  Page 2 of 5

| **Passage A**    Your doctor has sent you to have a (1) ____ X-ray.  a. stomach  b. diabetes  c. stitches  d. germs    You must have an (2)_____stomach when you come for(3)_____  a. asthma a. is  b. empty b. am  c. incest c. if  d. anemia d. it  The X-ray will (4)_____from 1 to 3 (5)____ to do.  a. take a. beds  b. view b. brains  c. talk c. hours  d. look d. diets | 1.  2.  3.  4.  5. |
| --- | --- |
| **THE DAY BEFORE THE X-RAY**    For supper have only a (6)______ snack of fruit, (7)______ and jelly, with coffee or tea.  a. little a. toes  b. broth b. throat  c. attack c. toast  d. nausea d. thigh    After (8)_______, you must not (9)______or drink  a. minute a. easy  b. midnight b. ate  c. during c. drank  d. before d. eat      anything at (10)_______ until after you have (11)______ the X-ray.  a. ill a. are  b. all b. has  c. each c. had  d. any d. was | 6.  7.  8.  9.  10.  11. |

**TOFHLA Page 3 of 5**

| **THE DAY OF THE X-RAY**      Do not eat (12)________.  a. appointment  b. walk-in  c. breakfast  d. clinic      Do not (13)______, even (14)______.  a. drive a. heart  b. drink b. breath  c. dress c. water  d. dose d. cancer      If you have any (15)________, call the X-ray (16)_______ at 616-4500.  a. answers a. Department  b. exercises b. Sprain  c. tracts c. Pharmacy  d. questions d. Toothache | 12.  13.  14.  15.  16. |
| --- | --- |

| **PASSAGE B**    I agree to give correct information to (17)______ if I can receive Medicaid.  a. hair  b. salt  c. see  d. ache    I (18)______ to provide the county information to (19)_______ any  a. agree a. hide  b. probe b. risk  c. send c. discharge  d. gain d. prove    statements given in this (20)________ and hereby give permission to  a. emphysema  b. application  c. gallbladder  d. relationship    the (21)______ to get such proof. I (22)______ that for  a. inflammation a. investigate  b. religion b. entertain  c. iron c. understand  d. county d. establish    Medicaid I must report any (23)________ in my circumstances  a. changes  b. hormones  c. antacids  d. charges    within (24)_______ (10) days of becoming (25)_______ of the change.  a. three a. award  b. one b. aware  c. five c. away  d. ten d. await | 17.  18.  19.  20.  21.  22.  23.  24.  25. |
| --- | --- |

**TOFHLA** Page 4 of 5

**TOFHLA** Page 5 of 5

| I understand(26)_______ if I DO NOT like the (27)________ made on my  a. thus a. marital  b. this b. occupation  c. that c. adult  d. than d. decision      case, I have the (28)_________ to a fair hearing. I can (29)_______ a  a. bright a. request  b. left b. refuse  c. wrong c. fail  d. right d. mend      hearing by writing or (30)________ the county where I applied.  a. counting  b. reading  c. calling  d. smelling      If you (31)_______ AFDC for any family (32)________, you will have to  a. wash a. member  b. want b. history  c. cover c. weight  d. tape d. seatbelt      (33)__________ a different application form. (34)_________, we will use  a. relax a. Since  b. break b. Whether  c. inhale c. However  d. sign d. Because    the (35)_______ on this form to determine your (36)________.  a. lung a. hypoglycemia  b. date b. eligibility  c. meal c. osteoporosis  d. pelvic d. schizophrenia | 26.  27.  28.  29.    30.  31.  32.  33.  34.  35.  36. |
| --- | --- |

**Neighborhood Characteristics** Page 1 of 2

| **Aesthetic environment** | | Strongly agree | Agree | Neutral (neither agree nor disagree) | Disagree | Strongly disagree |
| --- | --- | --- | --- | --- | --- | --- |
| 1 | My neighborhood is attractive | 1 | 2 | 3 | 4 | 5 |
| 2 | There is a lot of trash and litter on the street in my neighborhood | 1 | 2 | 3 | 4 | 5 |
| 3 | There are interesting things to do in my neighborhood | 1 | 2 | 3 | 4 | 5 |
| 4 | There is enjoyable scenery in my neighborhood | 1 | 2 | 3 | 4 | 5 |
| 5 | There is a lot of noise in my neighborhood | 1 | 2 | 3 | 4 | 5 |
| **Walking/exercise environment** | |  | | | | |
| 1 | My neighborhood offers many opportunities to be physically active | 1 | 2 | 3 | 4 | 5 |
| 2 | Local sports clubs and other providers in my neighborhood offer many opportunities to get exercise | 1 | 2 | 3 | 4 | 5 |
| 3 | It is pleasant to walk in my neighborhood | 1 | 2 | 3 | 4 | 5 |
| 4 | There are enough trees in my neighborhood to provide shade | 1 | 2 | 3 | 4 | 5 |
| 5 | My neighborhood has heavy traffic | 1 | 2 | 3 | 4 | 5 |
| 6 | There are busy roads to cross when out for walks in my neighborhood | 1 | 2 | 3 | 4 | 5 |
| 7 | In my neighborhood it is easy to walk places | 1 | 2 | 3 | 4 | 5 |
| 8 | There are stores within walking distance of my home | 1 | 2 | 3 | 4 | 5 |

**Neighborhood Characteristics** Page 2 of 2

| 9 | In my neighborhood, the streets and sidewalks are in good condition | 1 | 2 | 3 | 4 | 5 |
| --- | --- | --- | --- | --- | --- | --- |
| 10 | I often see other people walking in my neighborhood | 1 | 2 | 3 | 4 | 5 |
| 11 | I often see other people exercise (for example, jog, bicycle, play sports) in my neighborhood | 1 | 2 | 3 | 4 | 5 |
| **Safety from crime** | |  | | | | |
| 1 | I feel safe walking in my neighborhood during the evening | 1 | 2 | 3 | 4 | 5 |
| 2 | My neighborhood is safe from crime | 1 | 2 | 3 | 4 | 5 |
| 3 | Violence is a problem in my neighborhood | 1 | 2 | 3 | 4 | 5 |
| **Access to healthy foods** | |  | | | | |
| 1 | It is easy to purchase fresh fruits and vegetables in my neighborhood | 1 | 2 | 3 | 4 | 5 |
| 2 | There is a large selection of fresh fruits and vegetables available in my neighborhood | 1 | 2 | 3 | 4 | 5 |
| 3 | The fresh produce in my neighborhood is of high quality | 1 | 2 | 3 | 4 | 5 |
| 4 | It is easy to purchase low-fat products (such as low-fat milk or lean meats) in my neighborhood | 1 | 2 | 3 | 4 | 5 |
| 5 | There is a large selection of low fat products available in my neighborhood | 1 | 2 | 3 | 4 | 5 |
| 6 | The low-fat products in my neighborhood are of high quality | 1 | 2 | 3 | 4 | 5 |
| General Comments: | | | | | | |

**Social Cohesion (Sampson scale)** Page 1 of 1

|  | | Strongly agree | Agree | Neutral (neither agree nor disagree) | Disagree | Strongly disagree |
| --- | --- | --- | --- | --- | --- | --- |
| 1 | This is a close-knit or unified neighborhood | 1 | 2 | 3 | 4 | 5 |
| 2 | People around here are willing to help their neighborhoods | 1 | 2 | 3 | 4 | 5 |
| 3 | People in this neighborhood generally don’t get along with each other | 1 | 2 | 3 | 4 | 5 |
| 4 | People in this neighborhood can be trusted | 1 | 2 | 3 | 4 | 5 |
| 5 | People in this neighborhood do not share the same values | 1 | 2 | 3 | 4 | 5 |
| General Comments: | | | | | | |

**Neighborhood Safety** Page 1 of 5

| 1 | During the past six months, how often was there a fight in this neighborhood in which a weapon was used? | Often  Sometimes  Rarely  Never |
| --- | --- | --- |
| 2 | Any gang fights? | Often  Sometimes  Rarely  Never |
| 3 | A sexual assault or rape? | Often  Sometimes  Rarely  Never |
| 4 | A robbery or mugging? | Often  Sometimes  Rarely  Never |
| 5 | How safe from crime do you consider your neighborhood to be? | Extremely safe  Quite safe  Slightly safe  Not at all safe |
| 6 | How would you rate your neighborhood as a place to live? | Excellent  Good  Only Fair  Poor |
| 7 | Compare your neighborhood to others in your county? | Much better  Better  Same  Worse  Much worse |

**Neighborhood Recreation Facilities** Page 2 of 5

| **I would like to ask you about the following things available in your neighborhood. Please tell me if there are any of the following within a 20-minute walk from your neighborhood, and if so, the condition in which they are in:** | | |
| --- | --- | --- |
| 1 | Public Park | Yes No |
| 2 | Public sports field, basketball court, or tennis court | Yes No |
| 3 | Public pool or beach | Yes No |
| 4 | Schools, colleges, or community centers with recreational facilities that are free and open to the public | Yes No |
| 5 | Gyms, health/fitness clubs, or pools that you have to join and pay for | Yes No |
| 6 | YMCAs or YWCAs | Yes No |
| 7 | Bicycle path in the street or park | Yes No |
| 8 | Are there sidewalks in your neighborhood? | Yes No |

**Neighborhood Participation** Page 3 of 5

| **I am going to read you a list of organizations. Please tell me if you regularly join in the activities of these organizations with people in your neighborhood:** | | |
| --- | --- | --- |
| 1 | A neighborhood association like a block association, a homeowner or tenant association or a crime watch group | Yes No |
| 2 | Religious groups or charitable organizations | Yes No |
| 3 | Parent-teacher associations or other school support or service groups | Yes No |
| 4 | Youth organizations such as youth sports league or the scouts | Yes No |
| 5 | Clubs or associations for senior citizens or older people | Yes No |
| 6 | A labor union | Yes No |
| 7 | A professional, trade, farm, or business association | Yes No |
| 8 | Adults sports clubs or leagues or an outdoor activity club | Yes No |
| 9 | A literary, art, discussion, or stud group or a musical, dancing, or singing group | Yes No |
| 10 | Any other hobby club or society | Yes No |
| 11 | Ethnic, nationality, or civil rights organizations | Yes No |
| 12 | Other public interest groups, political groups, or party committees | Yes No |

**Neighborhood Problems** Page 4 of 5

| **For each of the following items, please tell me whether it is currently not a problem in your neighborhood, somewhat of a problem, or a big problem:** | | |
| --- | --- | --- |
| 1 | Trash or litter in the streets | Yes No |
| 2 | Noise from traffic, other homes, airplanes or business | Yes No |
| 3 | Lack of safety for walking around after dark | Yes No |
| 4 | Lack of places to go for entertainment (restaurants, movie theaters, cafes, bars) | Yes No |
| 5 | Poor traffic and road safety | Yes No |
| 6 | Lack of places to shop | Yes No |
| 7 | Vandalism, like people breaking windows or spray painting building | Yes No |
| 8 | Vacant lots with trash or junk | Yes No |
| 9 | Assaults, muggings, or burglaries | Yes No |
| 10 | Lack of trees or green spaces | Yes No |
| 11 | People who don’t keep up their property or yards | Yes No |
| 12 | No sidewalks or sidewalks in bad condition | Yes No |

**Neighborhood Problems** Page 5 of 5

| 13 | Problems with public services such as street lighting, garbage pickup, and police | Yes No |
| --- | --- | --- |
| 14 | Lack of public transportation | Yes No |
| 15 | People fighting or arguing | Yes No |
| 16 | People selling illegal drugs | Yes No |

| General Comments: |
| --- |

**Food Insecurity** Page 1 of 1

| ***I’m going to read you two statements that people have made about their food situation. Please tell me whether the statement was OFTEN, SOMETIME, or NEVER true for (you/you and the other members of your household) in the last 12 months.*** | | |
| --- | --- | --- |
| 1 | “This food that we bought just didn’t last, and we didn’t have money to get more.” | Often true  Sometimes true  Never true  Don’t know/Refused |
| 2 | “We couldn’t afford to eat balanced meals.” | Often true  Sometimes true  Never true  Don’t know/Refused |
| 3 | In the last 12 months, since (date 12 months ago) did (you/you or other adults in your household) ever cut the size of your meals or skip meals because there wasn’t enough money for food? | Yes No |
| 4 | [Ask only if #3 = YES]  How often did this happen—almost every month, some months but not every month, or in only 1 or 2 months? | Almost every month  Some months but not every month  Only 1 or 2 months  Don’t know/Refused |
| 5 | In the last 12 months, did you ever eat less than you felt you should because there wasn’t enough money to buy food? | Yes No |
| 6 | In the last 12 months, were you ever hungry but didn’t eat because you couldn’t afford enough food? | Yes No |
| General Comments: | | |

**The Summary of Diabetes Self-Care Activities** Page 1 of 1

| The questions below ask you about your diabetes self-care activities during the past 7 days.  If you were sick during the past 7 days, please think back to the last 7 days that you were not sick. |
| --- |

Diet

| 1 | How many of the last SEVEN DAYS have you followed a healthful eating plan? | days |
| --- | --- | --- |
| 2 | On average, over the past month, how many DAYS PER WEEK have you followed your eating plan? | days |
| 3 | On how many of the last SEVEN DAYS did you eat five or more servings of fruits and vegetables? | days |
| 4 | On how many of the last SEVEN DAYS did you eat high fat foods such as red meat or full-fat dairy products? | days |

Exercise

| 5 | On how many of the last SEVEN DAYS did you participate in at least 30 minutes of physical activity? (Total minutes of continuous activity, including walking). | days |
| --- | --- | --- |
| 6 | On how many of the last SEVEN DAYS did you participate in a specific exercise session (such as swimming, walking, biking) other than what you do around the house or as part of your work? | days |

Blood Sugar Testing

| 7 | On how many of the last SEVEN DAYS did you test your blood sugar? | days |
| --- | --- | --- |
| 8 | On how many of the last SEVEN DAYS did you test your blood sugar the number of times recommended by your health care provider? | days |

Foot Care

| 9 | On how many of the last SEVEN DAYS did you check your feet? | days |
| --- | --- | --- |
| 10 | On how many of the last SEVEN DAYS did you inspect the inside of your shoes? | days |

Smoking

| 11 | Have you smoked a cigarette—even one puff—during the past SEVEN DAYS?  (*If ‘No’, then form is complete*) | Yes No |
| --- | --- | --- |
| 12 | If ‘yes’, how many cigarettes did you smoke on an average day? | cigarettes/day |
| General Comments: | | |

**The 8—Item Morisky Medication Adherence Scale** Page 1 of 1

| 1 | Do you sometimes forget to take your diabetes medications? | Yes No |
| --- | --- | --- |
| 2 | Over the past 2 weeks, were there any days when you did not take your diabetes medications? | Yes No |
| 3 | Have you ever cut back or stopped taking your diabetes medications without telling your doctor because you felt worse when you took it? | Yes No |
| 4 | When you travel or leave home, do you sometimes forget to bring along your diabetes medications? | Yes No |
| 5 | Did you take your diabetes medications yesterday? | Yes No |
| 6 | When you feel like your diabetes is under control, do you sometimes stop taking your medicine? | Yes No |
| 7 | Do you ever feel hassled about sticking to your diabetes treatment plan? | Yes No |
| 8 | How often do you have difficulty remembering to take all your diabetes medication? | Never  Almost Never  Sometimes  Quite Often  Always |

**Charlson Comorbidity** Page 1 of 1

| We are asking you the following questions in order to determine your medical history.  Please respond to each question either ‘yes’ or ‘no’ | | |
| --- | --- | --- |
| 1 | Have you ever had a heart attack? | Yes No |
| 2 | Have you ever been hospitalized or treated for heart failure? | Yes No |
| 3 | Have you ever been told you have a heart murmur or valvular heart disease, or have you had a valve replacement? | Yes No |
| 4 | Do you cough first thing in the morning in the winter, and do you cough up mucus on most of these days? | Yes No |
| 5 | Have you ever had wheezing or asthma? | Yes No |
| 6a | Have you had pain in your calf while walking, especially when walking uphill or hurrying, making it necessary to stop or slow down? If so, is it relieved when you stop or slow down in <10 minutes? | Yes No |
| 6b | If yes, have you had a peripheral bypass operation for this problem or your leg operated? | Yes No |
| 7a | Have you ever had a stroke? | Yes No |
| 7b | If yes, do you still have difficulty moving an arm or a leg as a result? | Yes No |
| 8a | Have you ever had diabetes or high blood sugar? | Yes No |
| 8b | If yes, to 8a, has diabetes caused problems with your kidneys or eyes or with the feeling in your feet and legs? | Yes No |
| 9a | Have you had liver trouble such as cirrhosis or permanent liver damage? | Yes No |
| 10 | Have you had trouble with stomach ulcers? | Yes No |
| 11 | Have you ever vomited blood or passed blood in your stool or had colitis? | Yes No |
| 12a | Have you had cancer (except skin cancer)? | Yes No |
| 12b | If yes to 12a, did it spread to distant sites? | Yes No |
| 13 | Have you had any rheumatic disease? | Yes No |
| 14 | Do you have HIV or AIDS? | Yes No |
| 15 | Have you had a change in kidney function, dialysis or renal transplant? | Yes No |

**SF-12 Health Status Page 1 of 2**

| 1 | In general, would you say your health is: (choose one) | 1 Excellent  2 Very Good  3 Good  4 Fair  5 Poor | |
| --- | --- | --- | --- |
| The following items are about activities you might do during a typical day.  Does your health now limit you in these activities? If so, how much? (Check one box) | | | |
| 2 | Moderate activities, such as moving a table, pushing a vacuum cleaner, bowling, or playing golf | | 1 Yes, limited a lot  2 Yes, limited a little  3 No, not limited at all |
| 3 | Climbing several flights of stairs | | 1 Yes, limited a lot  2 Yes, limited a little  3 No, not limited at all |
| During the past 4 weeks, have you had any of the following problems with your work  or other regular daily activities as a result of your physical health? | | | |
| 4 | Accomplished less than you would like | | 1 Yes 2 No |
| 5 | Were limited in the kind of work or other activities | | 1 Yes 2 No |
| During the past 4 weeks, have you had any of the following problems with your work  or other regular daily activities as a result of any emotional problems (such as feeling depressed or anxious)?  (choose one answer for each line) | | | |
| 6 | **Accomplished less** than you would like | | 1 Yes 2 No |
| 7 | Didn’t do work or other activities as **carefully** as usual | | 1 Yes 2 No |
| 8 | During the past 4 weeks, how much did pain interfere with your normal work (including both work outside the home and housework)? (Check one box) | | 1 Not at all  2 A little bit  3 Moderately  4 Quite a bit  5 Extremely |

**SF-12 Health Status** Page 2 of 2

| These questions are about how you feel and how things have been with you during the past 4 weeks.  For each question please give the one answer that comes closest to the way you have been feeling. | | |
| --- | --- | --- |
| How much of the time during the past 4 weeks…(Circle one answer for each line) | | |
| 9 | Have you felt calm and peaceful? | 1 All of the time  2 Most of the time  3 A good bit of the time  4 Some of the time  5 A little of the time  6 None of the time |
| 10 | Did you have a lot of energy? | 1 All of the time  2 Most of the time  3 A good bit of the time  4 Some of the time  5 A little of the time  6 None of the time |
| 11 | Have you felt downhearted and blue? | 1 All of the time  2 Most of the time  3 A good bit of the time  4 Some of the time  5 A little of the time  6 None of the time |
| 12 | During the past 4 weeks, how much of the time has your physical health or emotional problems interfered with your social activities (like visiting with friends, relatives, etc.) | 1 All of the time  2 Most of the time  3 Some of the time  4 A little of the time  5 None of the time |
| General Comments: | | |

**Vital Signs** Page 1 of 1

| 1 | Weight: | **. .** lbs. |
| --- | --- | --- |
| 2 | Height: | . . in. |
| 3 | Pulse: | (Per min.) |
| 4 | Systolic Blood Pressure: | (mm/Hg) |
| 5 | Diastolic Blood Pressure: | (mm/Hg) |
| 6 | LDL: | (mg/dL) |
| 7 | A1C level: | . . % |
| General Comments: | | |
